# Incidence of lung tumours in LX mice exposed to (1) free radicals; (2) SO2.

**DOI:** 10.1038/bjc.1967.71

**Published:** 1967-09

**Authors:** P. R. Peacock, J. B. Spence

## Abstract

**Images:**


					
606

INCIDENCE OF LUNG TUMOURS IN LX MICE EXPOSED TO

(1) FREE RADICALS; (2) S02
P. R. PEACOCK AND J. B. SPENCE

From the Cancer Research Department, Royal Beatson Memorial Hospital, Glasgow

Received for publication November 30, 1966

Although the spontaneous incidence of pulmonary adenoma varies greatly
between line-bred strains of mice, they all show a higher species susceptibility to
this very characteristic tumour than other species of small rodent kept under
similar laboratory conditions. Moreover, some chemical carcinogens for mouse
lung, e.g. urethane and polycyclic hydrocarbons, induce similar tumours to those
of spontaneous origin, but at an earlier age and in greater numbers than are found
in untreated controls. Thus, the mechanism of carcinogenesis in the mouse lung
appears to be very complex, involving unidentified factors responsible for the
spontaneous tumours, in addition to controlled experimental factors.

A close association between the occurrence of subpleural alveolar hyperplasia
and neoplasia and the presence of engorged lymphatics filled with apparently
normal lymphocytes in the affected sector of the lung, has been reported (Peacock
and Peacock, 1966). While the significance of this association is not clear, it
might be of aetiological importance. Occasionally such engorged lymphatics are
seen in the normal subpleural lymphatic sites without any epithelial hyperplasia,
in apparently healthy lungs, and these may be regarded as physiological.

On the other hand, in our experience subpleural alveolar hyperplasia is rarely
observed in the absence of such lymphatic engorgement. Clearly, in a single
section or in a series of sections taken from a single lung, one cannot form an
adequate impression of the duration of such lymphatic engorgement; but it
seems possible that if it is maintained for more than a certain length of time,
alveolar hyperplasia of the associated overlying epithelium may follow automati-
cally, and this in its turn may provide a suitable site for the action of potential
carcinogens.

It seemed desirable, therefore, to devise experiments which would create areas
of subpleural lymphatic engorgement and maintain them for a known length of
time, and observe the possible effects of such treatment on the subsequent inci-
dence of hyperplastic and neoplastic lesions in the lung. With this in mind, we
chose two non-cumulative inorganic irritants capable of being administered as
airborne pollutants, namely free radicals and sulphur dioxide.

MATERIALS AND METHODS

Free radicals were chosen partly because they are present in cigarette smoke,
which is associated with cancer of the lung in man, and also because free radicals
are generated in the electrostatic air filter which was used in previous experiments
on the possible influence of airborne soot as a potential carcinogen for the mouse
lung (Peacock, 1962). Sulphur dioxide was chosen because of its presence in

EXPOSURE TO FREE RADICAL AND S02 AND LUNG TUMOURS

urban atmospheric pollution and because it is a gaseous and non-cumulative
irritant which is easily controlled.

Mice of the LX colony, bred in this laboratory from stock kindly supplied by
Dr. Bloom, were used because they are known to be highly susceptible to the
induction of lung adenoma in response to urethane, and because they are hetero-
zygous though selectively bred for large size (Bloom, 1964).

To house the experimental mice, two identical perspex chambers, each
measuring 50 x 40 x 85 cm. and of approximately 180 litres' capacity, were used.
These chambers were equipped with two perspex shelves, each of which could
carry four standard mouse boxes; 12 boxes in all. The chambers were closed by
sliding doors and had input ventilation tubes of 5 cm. diameter and similar output
openings on one side of each cage. No intercommunication was possible between
the two chambers.

The mice were segregated by sex and were kept in galvanised boxes each con-
taining not more than five mice, usually members of one litter. Food pellets
(Diet 41) and tap water in plastic bottles, were constantly available.

Group 1: 41 Male and 39 female, three-month-old LX mice born at about the
same date as the experimental groups, were kept in the main animal house in
similar boxes.

Group 2: 30 male and 30 female LX mice, three months old, were kept in
Chamber 1, which was equipped with thin copper sleeve electrodes, 1 25 cm. broad
and 6 mm. apart, fitted around the input ventilation duct. The lower electrode
was connected to the active terminal of a diathermy unit giving an output of
1.5 kw at 2 kv. The upper electrode was earthed.

The technique for free radical reproduction was that of radio-frequency
discharge (Shaw, 1960). The apparatus was switched on for 3 hours daily from
Monday to Friday inclusive throughout the experiment.

Group 3: 35 male and 30 female LX mice, three months old, were kept in
Chamber 2 under similar conditions to those in Group 2, except that the ventilation
duct was equipped with a side tube through which SO2 could be added to the input
air.

By preliminary pilot trials a rate of 20 ml. per minute for 5 minutes (500 p.p.m.)
was found to be well tolerated, and this dosage was given daily from Monday to
Friday throughout the experiment.

All animals were inspected daily by the technical staff throughout the experi-
ment, and sick animals were reported, and were examined by one of us (P.R.P.)
and if considered to be seriously ill were killed by chloroform and immediately
autopsied. Despite this, some animals were found dead and were autopsied as
soon as possible after discovery. Most survivors over two years old were killed
and immediately autopsied.

The procedure adopted was described in detail in a previous communicatioin
from this laboratory (Peacock and Peacock, 1966). Suspicious lesions in any
organ were processed for histology. The lungs were inflated with 1% formalin
and removed intact for further inspection. The heart was removed and all
obvious lung lesions noted. The lungs were then compressed between the lid and
inverted base of a plastic petri dish and examined from both sides by transmitted
light. In this way, lesions of less than 1 mm. diameter can be seen by the narked
eye as translucent spots. All such lesions were processed for serial sectioning,
stained by Van Geison or Mallory and Gomori's aldehyde fuchsin elastic stain, in

607

P. R. PEACOCK AND J. B. SPENCE

order to distinguish between alveolar hyperplasia and neoplasia, in whichi con-
dition the elastica is defective.

RESULTS

Only mice that survived for three hundred days or more are considered in
assessing the results because no primarv tumours of the lung were seen in LX mice
below this age. The essential details of experimental data, autopsy and histology
for each mouse, and the site and degree of neoplasia, when present, are given in
Tables I to III.

In order to give some numerical value to the degree of neoplasia in the lungs,
one (+) was awarded for each mm. of tumour diameter for the largest tumour in
any one mouse. A mouse with one tumour of 3 mm. and several smaller tumours,
scores (3), and so on. Admittedly this method under-estimates the differences
between a single tumour of 1 mm. or less diameter discovered during histological
examination of an apparently normal lung and a mass of tumours which caused
death or serious respiratory embarrassment. However, the method of attempting
to count individual tumours is thought to be less satisfactory because a number of
small tumours may become confluent and so the number of recorded tends to grow,
less as the tumours grow larger.

The incidence of primary lung hyperplasia and neoplasia is shown in Tables IVt
and V. Since the degree of neoplasia is debatable all neoplastic lesions are
included under Adenoma in Table IVr. (see Discussion). It will be seen that in the
mice exposed to SO2 there is an increased incidence of primary neoplasia in the
lung of both sexes, from 31 % to 5400 in males and from 17 % to 43 o in females.

In those exposed to free radicals there was an increase of approximately 1000
in males and 6% in females. Primary lung tumours in males occurred about twice
as frequently as in females in groups 1 and 2.

DISCUSSIONi

The anatomical and histological grading shows about equal distribution
between tumours of subpleural and other origin in males of groups 1 and 2. and
in females of group 3; in the other groups subpleural exceeded other-site tumours.

A number of factors which cannot easily be quantitated must be considered in
assessing these results. Thus. the grading of neoplasia as carcinoma or adenoma
is not always easy. Lung tumours in mice rarely metastasise outside the thorax
and even large tumours replacing a whole lobe may show no invasive tendency.
We classify as carcinoma only those tumours which invade blood vessels (fig. 6) or
other organs, though in many adenomata atypical growth suggesting malignancy
may be found.

For this reason all primary tumours of the lung are included under the heading
Adenoma " in Table IN' and some of these also appear under 'Primary Carci-
noma" and under " Hyperplasia ".

All the " Primary carcinoma  mice also had adenoma, but some  Hyper-
plasia " mice had no neoplastic lesions.

The distinction between hyperplasia and neoplasia of the pulmonary alveolar
epithelium is somewhat arbitrary since both types of lesion are often found together
or in different parts of the same lung. Inevitably therefore subjective judgment
plays a part in assessing the degree of neoplasia.

In general, the group exposed to SO2 had more and large primary lung tumours

60,8

EXPOSURE TO FREE RADICAL AND S02 AND LUNG TUMOURS

than untreated controls, and at an earlier age. Those exposed to free radicals
showed an incidence of lung tumours intermediate between the SO2 and control
group.

The occurrences of primary carcinoma of the lung in females was limited to
the S02 group. Fewer of the mice exposed to S02 were free from detectable
primary neoplastic lung lesions than in the other two groups. In many animals
several stages of hyperplasia and neoplasia were present together at autopsy.
In heterozygous animals, genetic factors may determine variation in the effects of
environmental carcinogens and might be held responsible for the higher incidence
of lung tumours in the SO2 group. Against this probability is the fact that
tumours of other organs, notably the liver in males and the lymphatic system in
females, show no such group distinction. In Table VI a comparison is made of
the actual and percentage incidence of hepatoma and lymphomatoses (including
leukaemia and lymphosarcoma), on the same basis as that used in Table V. For
ease of comparison the results for lung and liver tumours are shown in the form of
a histogram (Fig. 1).

90     1      2       3              1      2       3
80-
70-

50-
40-

30                             AA

A
10                       ~~~~~~~A

IH      H       H

Fsi(. 1. Histogram showing percentage distribution of pulmonary papillary adenoma A and

hepatoma H; histologically malignant tumours shown in black. Figures at head of columns
correspond with Groups 1, 2, and 3.

It is clear that there is no significant variation in the distribution of hepatoma
between the three groups, but that the incidence in the males is more than twice
that in the females. The distribution of lymphomatosis shows a greater sus-
ceptibility of females in all groups, but no apparent relationship to experimental
conditions. The higher incidence of lymphomatosis in male control mice may
be related to their lower incidence of lung tumours, but with the small number
involved (5/35) it is not thought to be aetiologically significant.

Independent statistical analysis of the figures in Tables IV-VI by our colleague
Dr. S. Iversen shows that such a distribution of tumours could be a matter of
chance.

609

P. R. PEACOCK AND J. B. SPENCE

o _       _

0~~~~

-. X   m;

s        -; 3   z

bO        (Dm

0

t    0 4 5

4 E .3 ;' g

4a 0

(+ I  I  I  I  I  I

I   I  I  I  I  I

,   CD  _>e

(D00 011

?te         MSC?4~~J

0 ~  ~   01

o C)   010 0oo

oZ m1 to oo 0

co _NCSC

__

i I

I +
I   I

I +

-1-
.00
-*

0 0

10 C-

CO-

I
I

el

0

0

0

01

10

P-

?
?

1-
-C

P-

co

10
P-

I      I        I
I   I      I

I      I      I
I      I      I

q*0001

1Coo _

_ _M C__

10 U I
_* _0 _

+
+

01
710

P-

0-

uOC

01
0l
10

+1

I   I
I   I
I I

> CO

41010

_4

00C
10 CO

+
I

0

10

CO

0

CZ

In

C*

P-

I        +
I 0 I'  I  toI

I  -     I -  -   0 x

0       1  0 10- -  00

1*10 m C* 10 -* 10010

-4 0 t- 1- 0000

100111111

CC

P.

1-4

HA

_  _  _ -~ !4e

I?I?I?

I_I_I_

I I I I
I I I I

I

+

r-

to

-

0
10

0

104

I   I

__

I I

I +
I +

00

co t-

10 cO
CO CO

'IO 01

1o10

+

01
0

I

t-

0

10

t-
ec

10

1-

i   I
I  I
i  I

I +

- C

I- -

4 CO

U: t-
cq C

CO 10

1_ 0

O     CO CO
CO CO - kt.
U- t- CO Go

10 e Ci CO

1100 0 0
1010 0101
"q P4 CO4 CO

610

EXPOSURE TO FREE RADICAL AND SO2 AND LUNG TUMOI

.   ;)  0      -  0 O

JRS

,--

-4

b
n
I  a)
II"

0

0-

a4

o. -_

r.>

la;

I _ -

r O

m

bE
0

0

0
~o
._

.-,

?

P-
0C
0

4 'q

X Ci

,--q  -   14-- 0 1 a

6> - -* - P- O O

*0

.     o   1.1   ce _d   CoDo

o     _  _  _  _  _

z)

S  -  ---

01  -o 1 c -410 e        01Cc _  -  *   allo1al o   1 0o  i

CO  .41i  co=   c    cOC  010  1 e10  101  10 to  1C  10  CI cc

_   10 I_  to    _ 10_4   *         __10  _I"4 41  10t c1  C  10

P- P- -  P-4 - -  P-4- -4 P- -4 - P- P- -4 r-- P- ~ P- "- r-

0
C.)
$14

I I 1I1I+    I
I   I   I   I   I   I  i

I   I   I   I   I   I  +
I   I   I   I   I   I  +

P-

C=1

t   =  e c: _  a0

co ce t- = -+  m

m  m  -  *1   o i

I         I
I         I
I          I
I          I

I     I      I
I        I   I
I     I    I

Il+

C10    COt-4 0

1 0o  1 0 00
U:    to to to

.~ .

I     I   I

I I   I
I   I   I
I   I   I

to CZ co

l4 l4 l

I        I
I        I
I I
I I

I +
I I
+ I
+ I

I       I
I       I
I I
I I

I             I            I
I             I            I
I             I            I
I             I            I

I      I
+       I

I      I
+ I

CO     * I41* l*Lit -  010   CM
COw  COC   COC   cOCOC   CO   C  1-

.0

0  1  040

o 4; c  .

4 50    F- ?4Ob )

o~~~~

0~~~~~

I  I 11 -tv

0

I  I  I  E

0  -4a~~~~~~~1

I    0

o

.~~~4  0 -   4

o

0

0
0   0

C)
CIO     0
0  c)~~~~

CO Ct  m

xo 10  *E
E4 4  '-  *

14-

611

00101010o 10o 100     10010    00 0    0C ooo   01    1 to  lat-  c  e0  0
E C9 10C c' d  e* c01  I0  101 to 4*"10  10  01ic    't I'*  10 10 1o  0

.-!?   --'?   --?   .14   ---?   .41,          --!?    --!?     --?    --!?   ---?   --'?    -t?   --?     --!?   -!?      -?     -!?     --!?    -0?      -,!?   --!?   -!?              --!?       S

P. R. PEACOCK AND J. B. SPENCE

_~   ~   C *       .

=   X     im gCs2U

0  0   0

0  ~~~~~~~~~~  ~~~~~~  Z~;.

o  * ~ ~ ~  ~  ~     0~~
w  ~~~~~ ~~4

CZ -

CoN

-o Co
"10

lf o

C= e9

10

eq Co
CO C

ot0

CO  _

CO s

Nd 10

COD -4
CO CO
eq eq
COi C

I I+

00 0 C

ko o o

Cq eq Co
CO CO CO

ce e a

_- _~ _-

I

-4

+

710

CA
10
CO
co

-4

xo

eq

.-

+
+

10
eq
10

0

"-

to

10

"-

el
CO

10
Co
CO
10
CO

w

CO

m

! I
I I

I +
I +

CO

to co

CO s
Co CC

to co
co CD

eq s
el CO
e.4 -.1

all e
_- _
10 co
CO* C

I
I

0

-C
-C
z
-ld

CO
CZ

0

eq

co
COt

+ I 4-

-f 1+
+ I +

eq eq

CO C Co
o Co to

co co N

Co C 10

CO eq 1

- CO -
to eq Co
CCO

P."  CX .

612

m
._Li

0-.

00
?4

*D
C.)~

H

* F:

;4    N

eq

I I

I +
I +,

I-

It-
CO

oo10 N

mm

-10
CO 1

t O

1- m
km cq
m m
10 eq4

+
+

Zq

eq
eq

I-

0
10
Co
CO

-4

ti-4

0  d.

(- gD -

0              1

N
-4- >,e      -

eq
o   ..   _z

w eq
O _

I I I +
I I Il+
I I I +
I I Il+

~-C
II-

COb C

Co eq eq CO

o N- N- N

O t- t- c

C- 1- C- -

10 114 10 CO

eq co o 10
CO cO CO CO

1- P- -4 -

1-
at

1-
oo

Co
N

0

In

10
CD

+
+

*II

1-

1-

N

CO

1-0

CO

eq

CO4

Il+

CO

NO
CO CO eq
N rN

N N 0
N N CO~

CO CO CO

- t- P-
i  koa
m  m c
,--   "-I  "-

I I
i I
I I
I I

.   .~ C4-

0

bOD

f0  + 4
;4

P4

tx

0C

I  .
0

EXPOSURE TO FREE RADICAL AND SO2 AND LUNG TUMOURS

0  ~~~~~oC3 0

0  ~ ~     ~   ~  0

.0 0    Ca~

E o ~ ~ ~ ~ ~ ~

_       ___                ? _     ?

Ill   III  I -i-?  ?  I I III   liii   1111     II
I   I   III   III   I I    I I ?-I-- III  1111  1111    II

I   III   III  I      I I I ill     liii   1111     II
I   III   III  I ?    I I -1- III     III  141+     II

01                    -         -                - CO

0       )

C O *0

ce~~

bC   1    1

(D  C    1

e1))      OO4     01 m= 4   aq -d  t7-  CO  C  0 CO10 = C O- to
kr- = .  0 1440  1n0  CO     C      CO  01  COOC =    =co -
COCO.44   - 11441  ~100N  f  CO  CO  CO   COCOC    COCO-

OCOI - C)COCO

114 1  414  114 in   0

-    -   -4  1-4 0101q

1 0 1 0n 10  0 1 0 1 1 0t
COCOC    COCOC

10    co        10   t-

(I-I C       C)    CO

01l    01       10    10
C O    C O      C O   C O

I-
01

CO
CO

01

CO

1- 1- 1-

in10 in

CO -4 C)4

100 110

COi COl CO

1- I- I- I-

10 CO C) 00
to CO CO to

0 1 1 0 P-   4   C O4

CO 1- 1-10f
CD t- t- CO

10 1- 1'- CO
1- r- 1- 1-

10   0Id Id

0101001e
CO CO to CO

1- r-     0>    10    I-
101 to         it     C
k- I-    I-     1-    I-

I- I-

I- CO

0101l

..* L

CO co

1010o
CO CO

01 m

0 1   0 1

01    01

CO    C O~

01

CO

410.

I10

CO

01

CO4

613

o~

0 0

Cs

A-   I

ft >

o     1

CO
0
CO

4
I

P. R. PEACOCK AND J. B. SPENCE

I +    II
I I   I   I   I
+   I+     I I
+   I+     I I

_q    _

10 OCO 00o
01 b C0 C oO
Ck CO     + 0

C -
10 k4

t _o

CO CO

ce  4P-

1010

- Co

,o u:

_ C"

10 1

PA

9z
0
0
._o

__

IC

0

Ca

;>

OD

I    I

I   +
I    I

I   +

m
CO

-    C

10  in

:    ?

4.)

Id
: .;

, 0-

i _

) ?

'D _

CO

0
no

_   _ _

1 +1

1 10+

CO- CoO

0??t

=  -

bCO

?
rj0   I

L -?

--  4

CO

_

-
+
CO

I I
I I

I +
I +

Coo CO

X cq

CO 1-
10 10

o) o

aq

01 CO
- 10
I" CO

+

I

I

0

0

1-

I

CSI

-4

01i

0

CO

0
O

cq

P-
ce

_____

I I  I  l+I
I  I  I  I   l

oomm
C4O o- -o

CO 00 CO 10

10100100>

Co 1 CO F 0
0cO 10 et CO

00000

10 CX 01 4 CO

101 ko -10
CO CO CO CO CO

_+
I +
I +

1+
I +

10

010

10 -

0o-4
t.- t-.

10

CO 10

t- 10

CO CO

cs4 o-

0

ce

0

Co

10

10

CO4

+

+

+

+

0

510

Ci

1-

CO

P-I

-.
1
C

1

co
1-

t-:
Ct

+
I

1-

-

1:

-

0
ko
CO

10
10

CO4

CO

to
to
e0

uz

P-
m

o

CO
0
0

*4
10
10
10

CO

4

m

CO

0
CO

00
to
oo

00

CO

oo
CO

-4
1o
CO

0
0

-1

c1

CO

0

to

1-

CO

cq
t-

01

CO4

cr; _

010

CO CO

CO CO

o Ot

0m10

C C

_- _et

0-4
P--4
PA
?4
pq

E--l

EXPOSURE TO FREE RADICAL AND S02 AND LUNG TUMOURS

,6 -  .  m  .o  .0  T      54 m      ..  - m

+5yt       0 R s XY j  9  t  e  ,,  ee .

I?  I II I II  I  I I  I 1+  I I  I  II   I  I I  +
I  1 1   I  I  I  I  14 I  I  I  Illt- I I I I ! II

+  I II  +- I+- +I+i I-I-II +   +  I+ III

-j      0  CO  _  _  _  CO - 1          -

_        t  14 c ff _ _ _ o _ _ t1q

_         _ _  _  _ _

- v   -   C O   C O -  C O

0-101_   -1   CO   C O OO '~   10 1

km 0        CO   0  CO O C
CD    C C   C O  4 f1 0

CO -10 s
Cf l Go _c_
a o 0-1 CO C

CO CO COi COl
.ce ce4 aa4 a

1o

Ci
Ct

P-

i-   O      -0   CO    O CO    O   0-I
O    C    1010   10 U:     m 0     t-
10       10 1  10   10 10    CO   CO

0
CO

ff4
10

CO

P-

U:
C*

10
CO

10
CO

CO
IC)
P-

-4 oa
co co

O 10
10 CO

10 -

O 00

0 '-4

C co

CC
CO
10
co

-

0

CO

c co

C) 10

10) 1*
CO CO

CO t
0-1 CO
10   -
CO CO

CO
0

CO
0
10
co

P-

t-
xo
1-
10

.-I

CO

1-

c00      Co     -_     CO     c   co

0       co       1*         10 101 0

C so    CO       t-   CO    k t- --

LO co
10 CO
t-* t-.

1010
0-1
CO CO

-

1q
0
CIO

0-
t-

10

CO4

CO

t- t- t-
co 1010O

10 10 10

CO CO co
U-4 P4 "e

615

54  1  5-

0

x      0

0

I   I _ _
I I   I   I

I+i   I I
I+   II

0

=O - t- O
1001  1 00

_ e1
-

t.- 1-

1q00
ff oO
CO CO

_fl _

0

0

4

0

bO

to

m

0._

?

54

54

m

0
C)

5-
0

a4,

05

bo

tr

0

(D
4

0

'.

ID t
| 04

04!

O6 o
CB

0

54
~ _

02 CB

.a      0

oQ o
D

*    s

0_ t

I _

o     I
I       I

O 0o e- e-

z a N      _-  -

0 C* C* a*

F- _  _

CO
0C

W-

Ct

1- =

C O

CO CO
0- CO
O CO

4 _4

C0 CO

1 _
CO CO

t
I
1:
t4-
c
a

E
c

.E
r-
c11
I"!
a
c
I.c
I

P. R. PEACOCK AND J. B. SPENCE

TABLE IV.-Actual Number and (%) of LX mice over 300 Days Old With or Without

Primary Lung Neoplasia or Hyperplasia

No lung       Primary

lesion      carcinoma    Adenoma    Hyperplasia
Group 1 (Control)

6 24/35 (69%) . 2/35 ( 6%) . 11/35 (31%) . 3/35 ( 9%)
9 23/30 (77%) .            5/30 (17%)  . 3/30 (10%)
Group 2 (Free radical)

d 16/29 (55%) . 3/29 (10%) . 12/29 (41%) . 4/29 (14%)
? 22/30 (73%) .             7/30 (23%) . 3/30 (10%)
Group 3 (Sulphur dioxide)

6' 13/28 (46%) . 2/28 ( 7%) . 15/28 (54%) . 5/28 (18%)
y 16/30 (53%) . 4/30 (18%) . 13/30 (43%) . 3/30 (10%)

TABLE V.-Site Incidence of Tumours/Mice at Risk

Subpleural    Other sites
Group 1 (Control)

6'. 9/35 (26%) . 7/35 (20%)

5/30 (17%) . 2/30 ( 7%)
Group 2 (Free radical)

6'  10/29 (35%) . 10/29 (35%)
?    6/30 (20%) . 3/30 (10%)
Group 3 (Sulphur dioxide)

6'  14/28 (50%) . 9/28 (32%)

10/30 (33%) . 10/30 (33%)

TABLE VI.-Actual Numbers and (%) of LX Mice Over 300 Days Old with Hepatoma

and/or Lymphomatosis

Hepatoma    Lymphomatosis
Group 1 (Control)

6   6/35 (17%) .   5/35 (14%)

2/30( 7%) .    8/30(27%)
Group 2 (Free radical)

6'. 6/29(20%) .    1/29( 3%)
?    2/30 ( 7%) .  9/30 (30%)
Group 3 (Sulphur dioxide)

6   5/28 (18%) .   1/28 ( 4%)

3/30 (10%) .   4/30 (13%)

EXPLANATION OF PLATES

FIG. 2.-6' LX 1317/2, age 96 days.  Died after accidental exposure for 5 hours to S02, 500

p.p.m. Lungs showed patchy congestion. H. & E. x 100.

FIG. 3.-Same mouse as above marked are enlarged. A small branch of bronchial artery

showing oedema and periarterial lymphangitis. H. & E.  x 350. (Nuclear detail empha-
sized by red filter).

FIG. 4.-V LX 1320/5, age 593 days; S02, 508 days. Killed in poor condition. Depressed

sternum; multifocal tumour (5 mm. diameter) in base of right lower lobe. Section shows
adjacent foci of alveolar hyperplasia (R) and papillary adenoma (L), separated by com-
pressed alveoli and lymphatics engorged with lymphocytes.
Mallory and Gomori's aldehyde fuchsin. x 85.

FIG. 5.-Enlargement of above, shows intact elastin in hyperplastic focus (R) and defective or

absent delastin in neoplastic focus (L). Note lymphatics engorged with apparently normal
lymphocytes and absence of other leucocytes. Contrast with Fig. 3. x 250.

FIG. 6.-? LX 1318/4, age 403 days: SO2, 318 days. Killed in poor condition. Right side of

thorax distended by huge tumour in right upper lobe compressing other right lobes and
displacing heart and depressing and invading right dome of diaphragm. No remote meta-
stases found. Section shows anaplastic papillary adenocarcinoma invading venule. H.
E. x 250.

All above photographed on Pan F film with green filter.

616

BRITISH JOURNAL OF CANCER.

2

3.

Peacock and Spence.

26

Vol. XXI, No. 3.

BRITISH JOURNAL OF CANCER.

4

6

Peacock and Spence.

Vol. XXI, NO. 3.

........

...

W         :

...

... % .

EXPOSURE TO FREE RADICAL AND SO2 AND LUNG TUMOURS     6

However, tumours occurred earlier in both experimental groups, the incidence
of lung tumours was about tw ice as high in mice of both sexes exposed to SO2 as in
controls, and in the females there were as many malignant tumours in the SO2
group as there were adenomata in the controls.

Malignancy was observed only in the larger tumours and presumably occurred
as a progressive development in a tumour which had already reached a diameter
of about 5 mm., and by implication had been growing for some time. From
the presence of such large tumours in the SO2 group, generally at an earlier
age than in the controls, it is concluded that the experimental conditions acce-
lerated the onset of neoplasia in susceptible mice. The results are consistent
with a positive effect of exposure to So2 and to a lesser extent, of exposure to
free radicals.

Thus it appears that the LX population contains a resistant moiety of about
5000 which is unaffected by the experimental conditions.

In the controls about 7000 of males and 8000 of females live to old age without
developing spontaneous lung tumours while the remainder show varying sus-
ceptibility.

How does the chemically simple gas SO2 induce primary lung tumours in
susceptible mice?

Early pilot experiments showed that with toxic exposure to SO2 death might
occur within an hour or two from acute oedema and congestion of the lung. In
less severe cases which survived for several days or were killed at various intervals
from hours to weeks after exposure, the most constant features were inter-alveolar
congestion and oedema and intra-alveolar exudate accompanied by lymphangitis
of subpleural and interstitial lymphatics. In the early acute inflammatory stages
many polymorphs were present along with lymphocytes in these lymphatics
(Figs. 2 and 3), but in later stages apparently normal lymphocytes persisted in
the engorged lymphatics, unaccompanied by evidence of chronic inflammatory
reaction and particularly in immediate juxtaposition to areas of alveolar
epithelial hyperplasia and neoplasia (Fig. 4 and 5).

It has been observed previously in other experiments that such lymphatic
engorgement is found fairly regularly associated with subpleural alveolar hyper-
plasia and neoplasia (Peacock and Peacock, 1966). This association of lymphatic
engorgement with local alveolar hyperplasia and neoplasia has not vet been
explained. It might represent a defence reaction which, in these cases, must have
failed to prevent progressive neoplasia; or it might be an aftermath of in-
flammatory lymphangitis which favours, in some way, local hyperplasia of adjacent
alveolar epithelium.

It is suggested that clinically subtoxic exposure to SO2 causes such lymphatic
engorgement and associated alveolar hyperplasia which, in the mouse, appear to
predispose to further progression to neoplasia (Fig. 4 and 5). The action of free
radicals is inconclusive.

It is concluded that the increased incidence of primary lung tumours in LX
mice of both sexes in Group 3 is a consequence of the initial essentially inflam-
matory reaction to SO2, followed by a state of apparent tolerance, which accele-
rates the inherent tendency of these mice to develop lung tumours spontaneously
but does not justify the classification of SO2 as a chemical carcinogen as generally
understood.

617

618                 P. R. PEACOCK AND J. B. SPENCE

SUMMARY

Three comparable groups of LX mice of both sexes were examined for primary
lung tumours and other lesions. Group 1, untreated controls; Group 2 exposed to
inhalation of free radicals; and Group 3 exposed to inhalation of S02.

No lung tumours were observed in mice below 300days of age and only those
which survived this age are considered in assessing the results.

An increased incidence of primary lung tumours in both sexes exposed to S02
was approximately doubled as compared with controls; carcinoma of the lung in
females was observed only in those exposed to SO2. There was a slight increase in
lung tumours in both sexes exposed to free radicals. The incidence of hepatoma
and lymphomatosis, the next most frequent tumours in controls, was unaffected
by the experimental conditions. There was an association between persistent
lymphatic engorgement and alveolar hyperplasia and the development of pro-
gressive, neoplasia, papillary adenoma and carcinoma, in the lungs of mice in all
groups. Repeated exposure to S02 apparently accelerated the unexplained
sequence of events which leads to the growth of spontaneous lung tumours in the
mouse.

REFERENCES

BLOOM, JOYCE, L.-(1964) J. natn. Cancer Inst., 33, 599.

PEACOCK, P. M., AND PEACOCK, P. R.-(1966) Br. J. Cancer, 20, 127.

PEACOCK, P. R.-(1962) in 'The Morphological Precursors of Cancer', Proc. Internat.

Confr., Perugia 1961, p. 605.

SHAW, T. M.-(1960) 'Formation and Trapping of Free Radicals' p. 47. Ed. Bass,

A. M. and Broida, H. P. Academic Press, Inc.

				


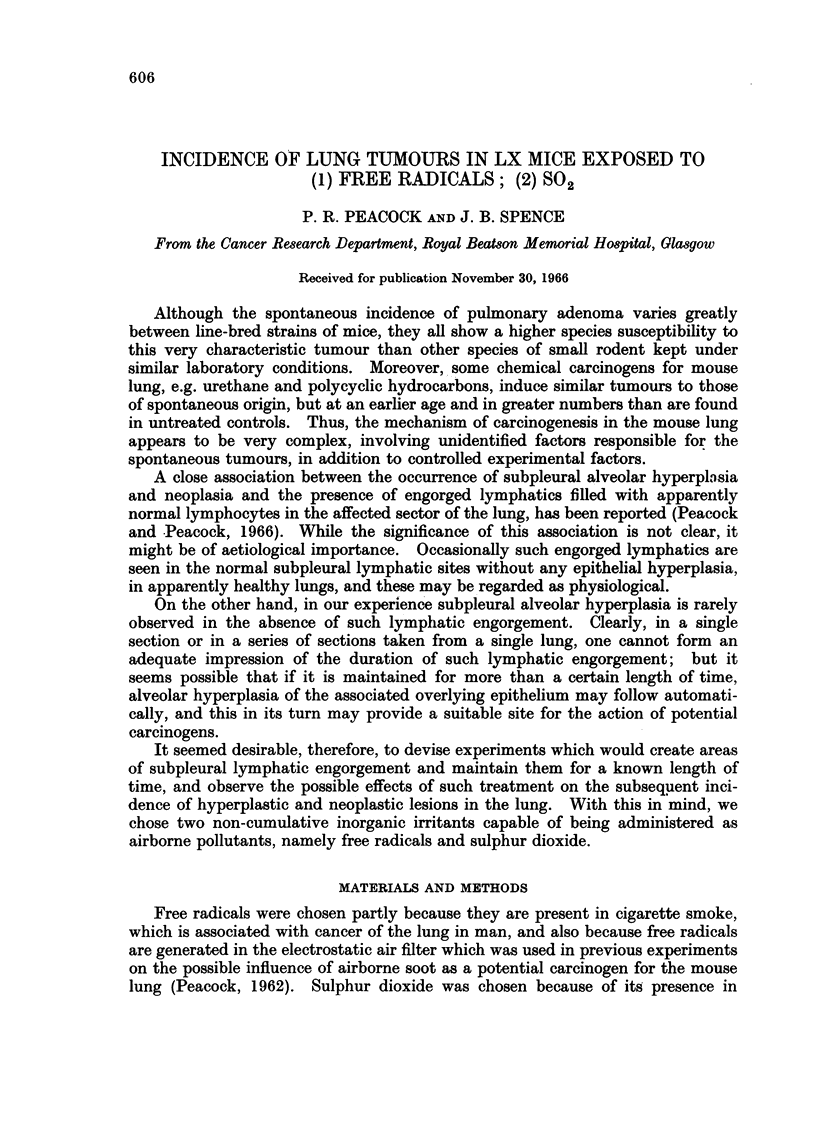

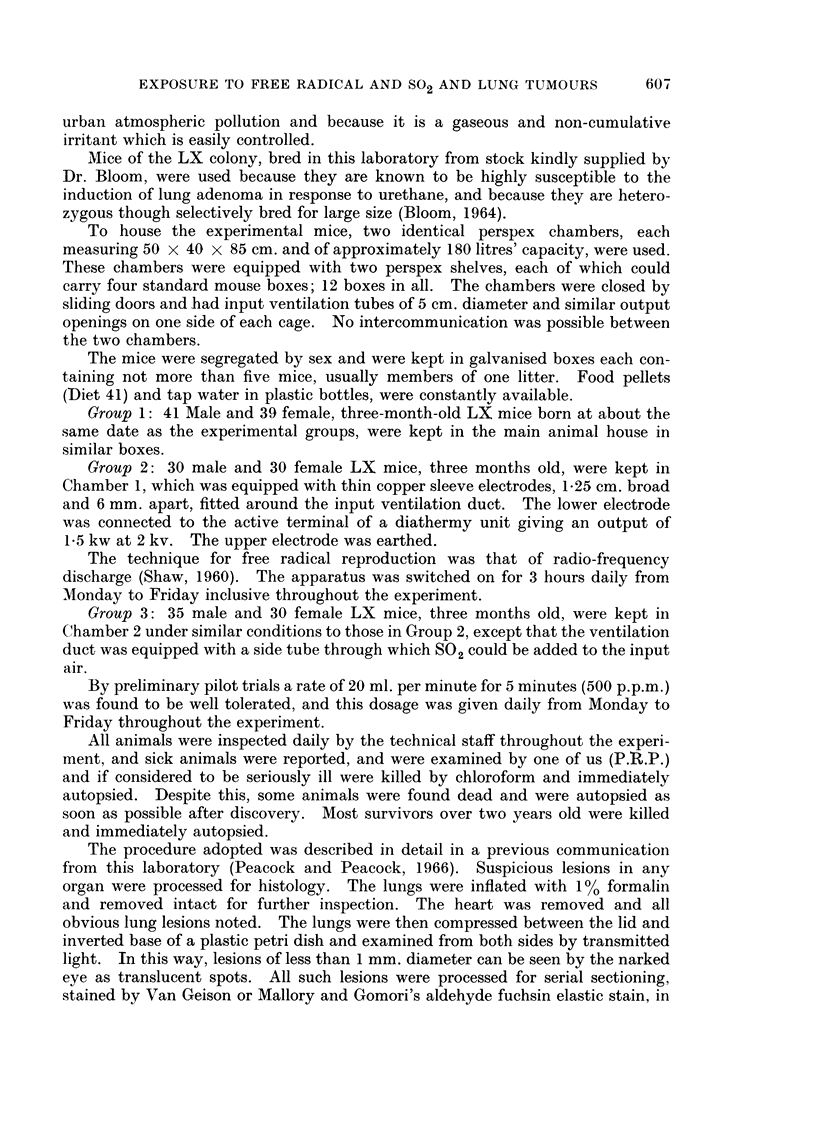

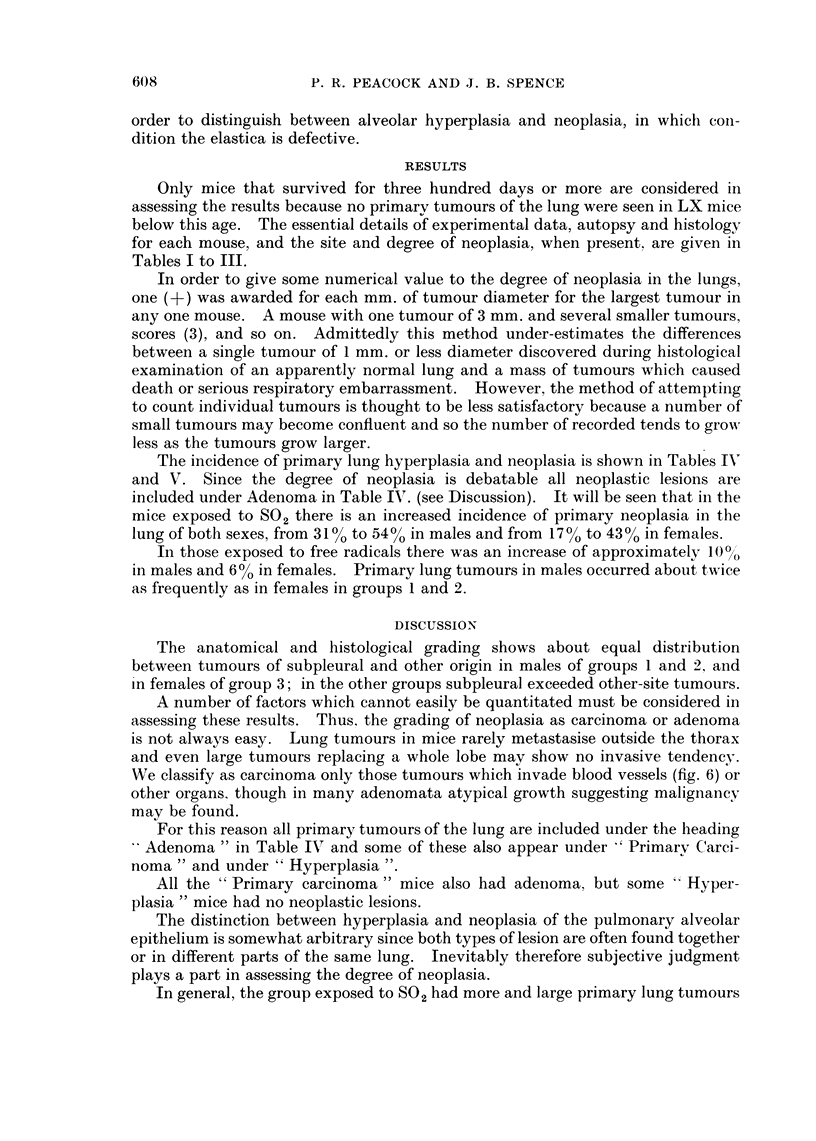

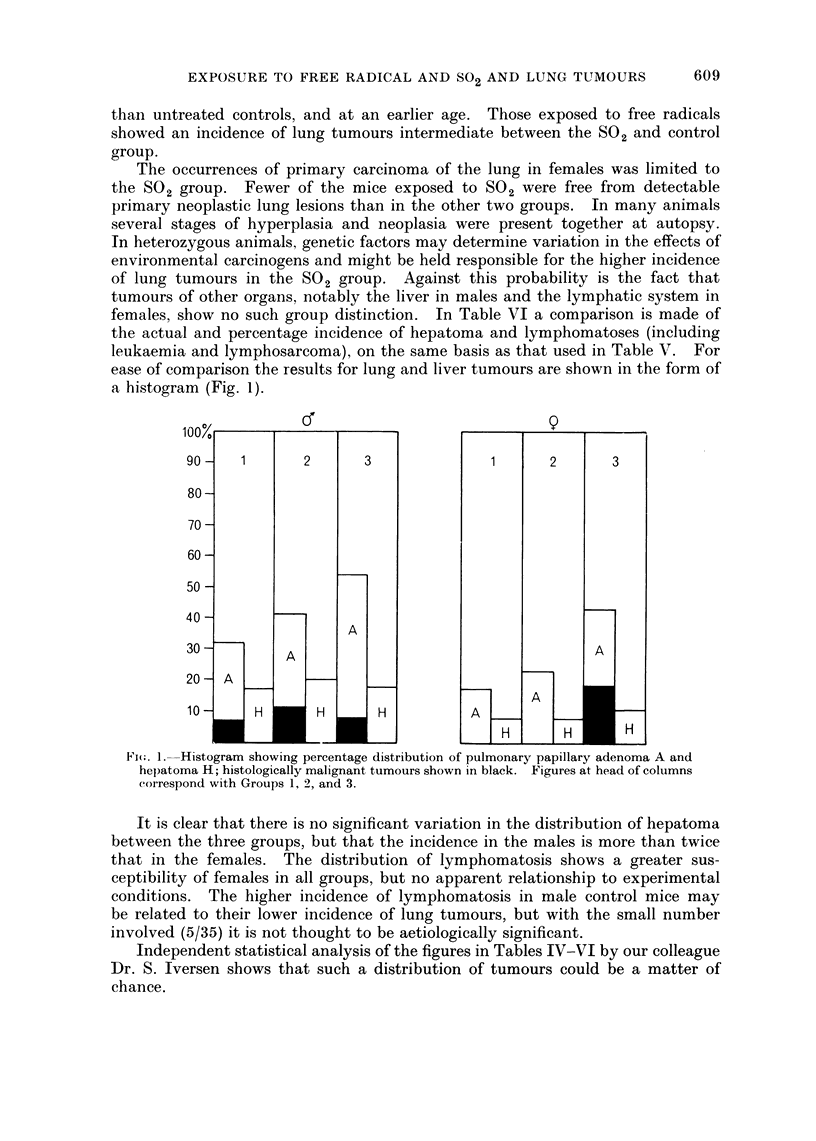

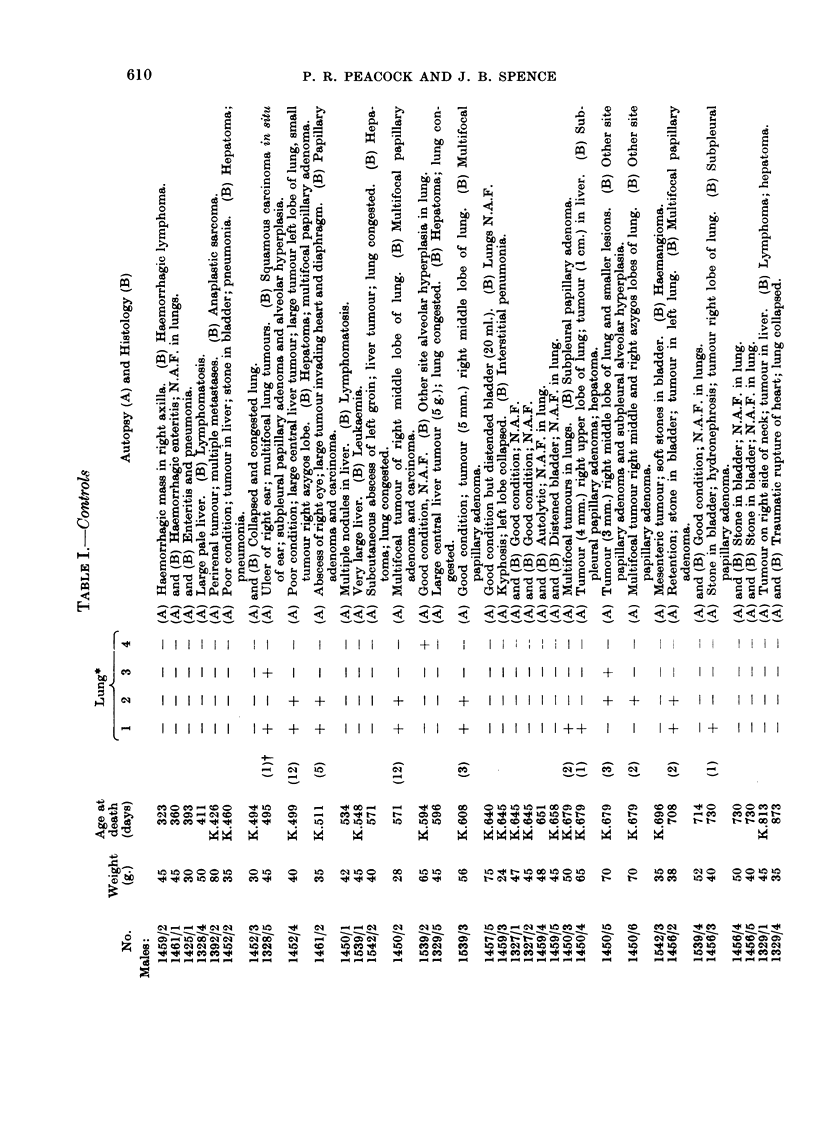

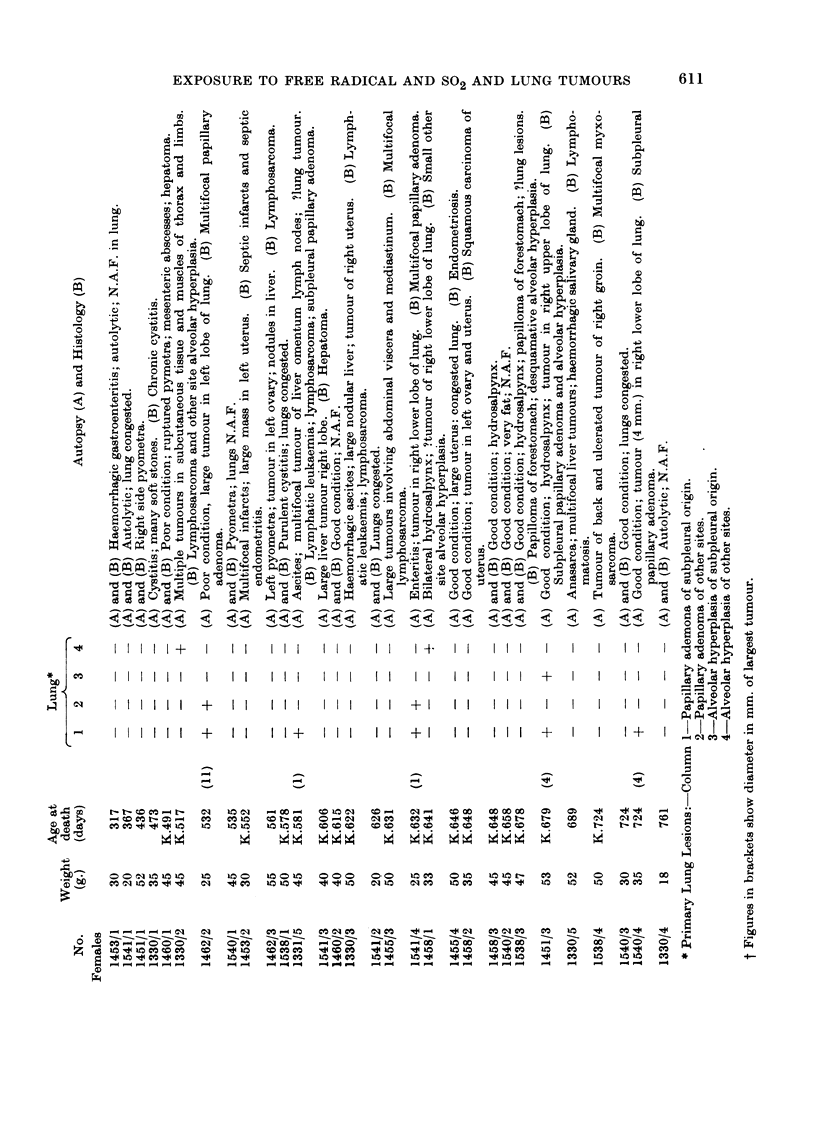

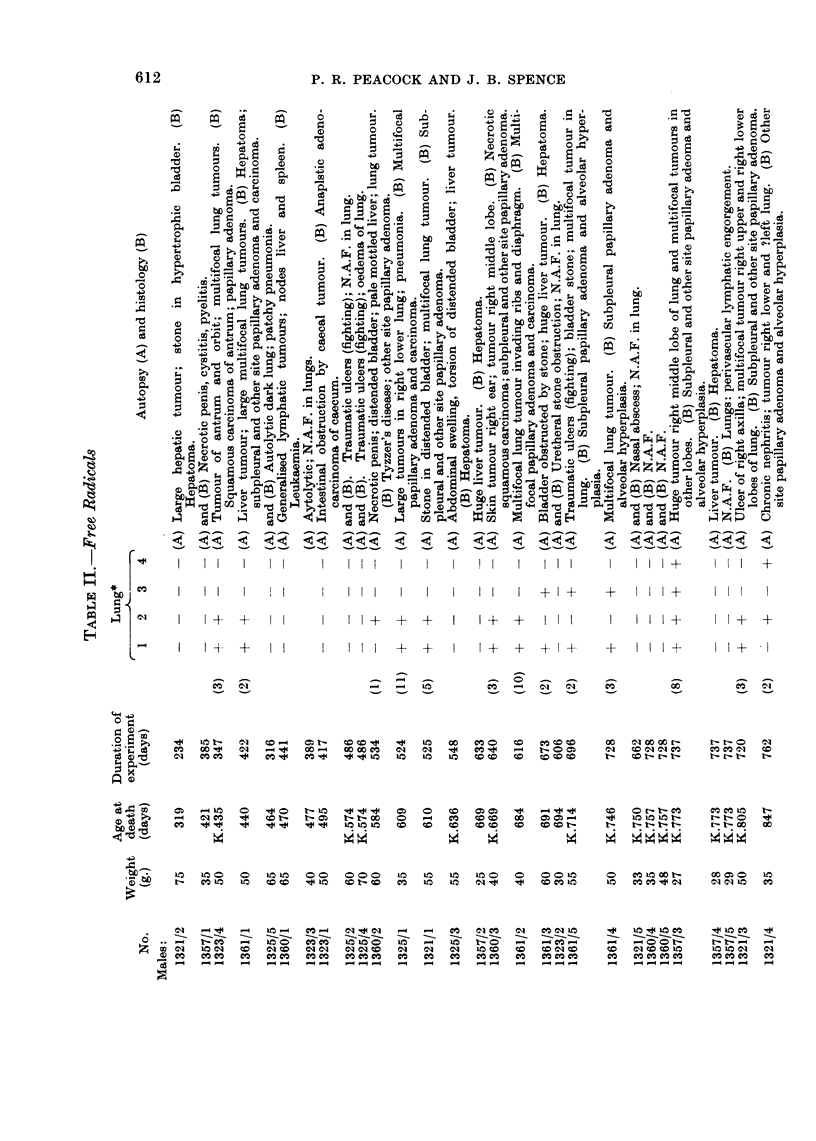

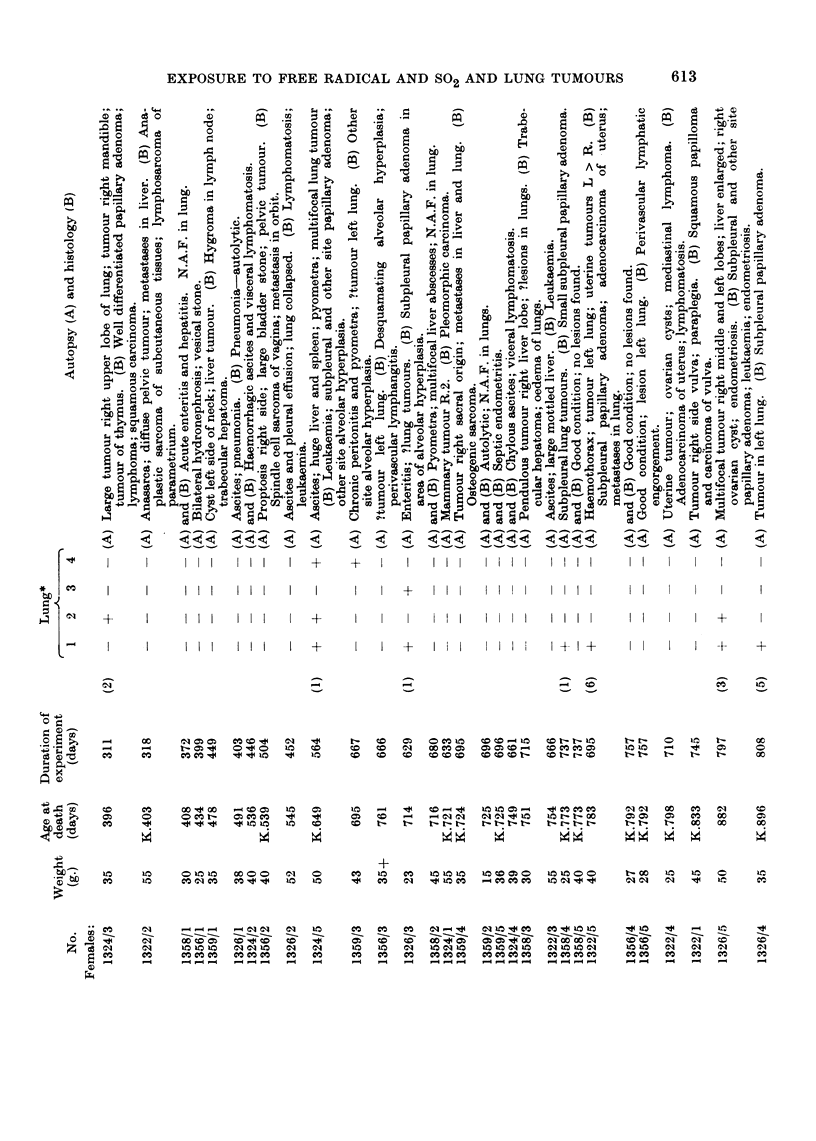

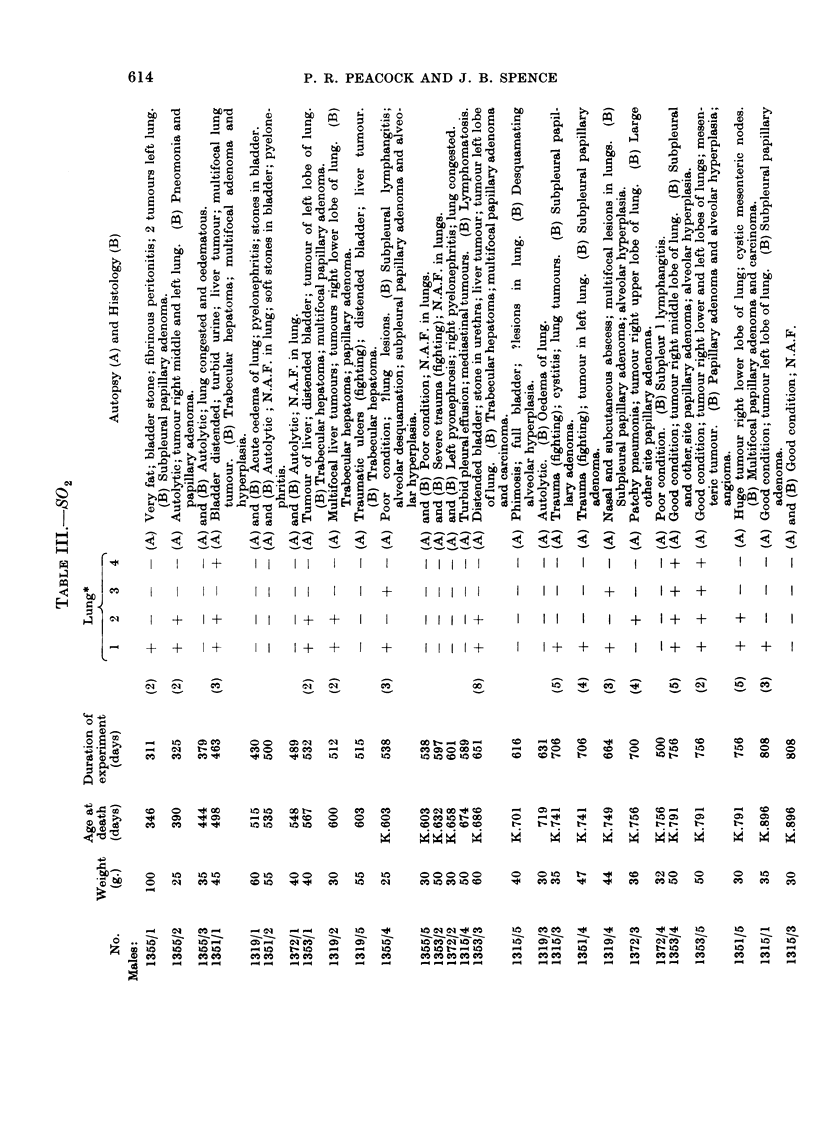

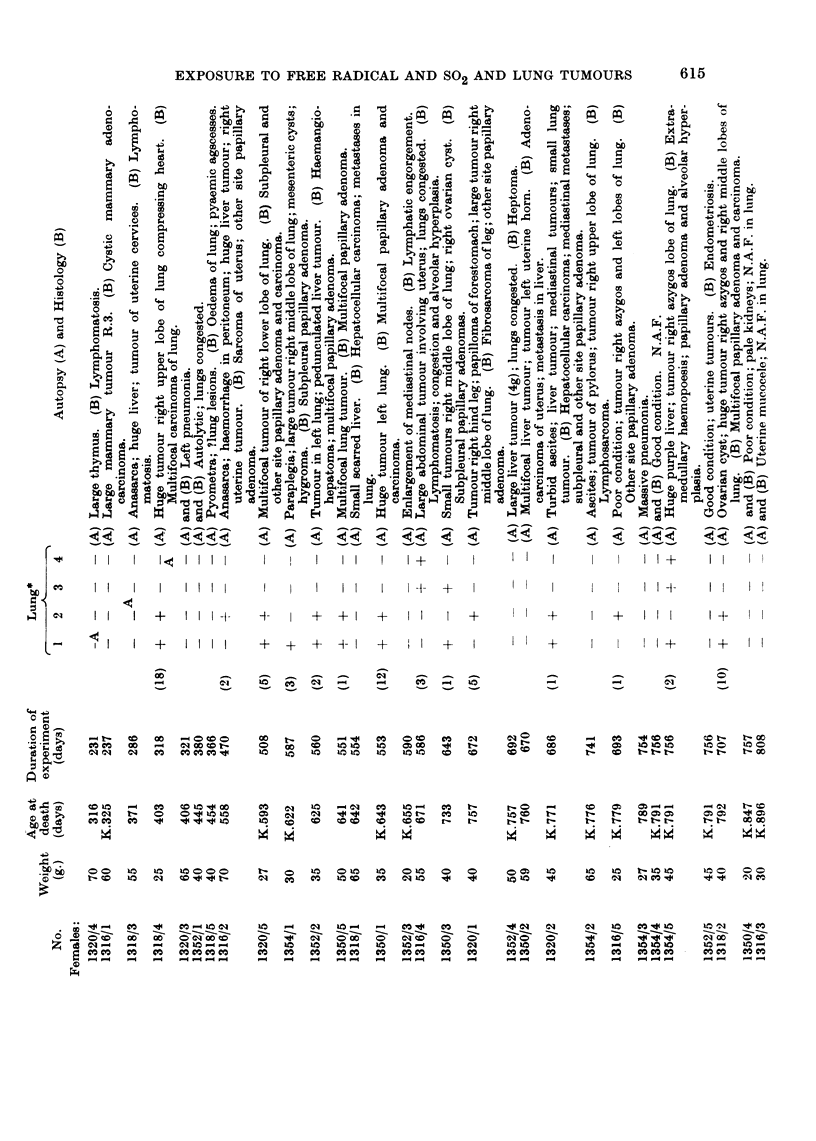

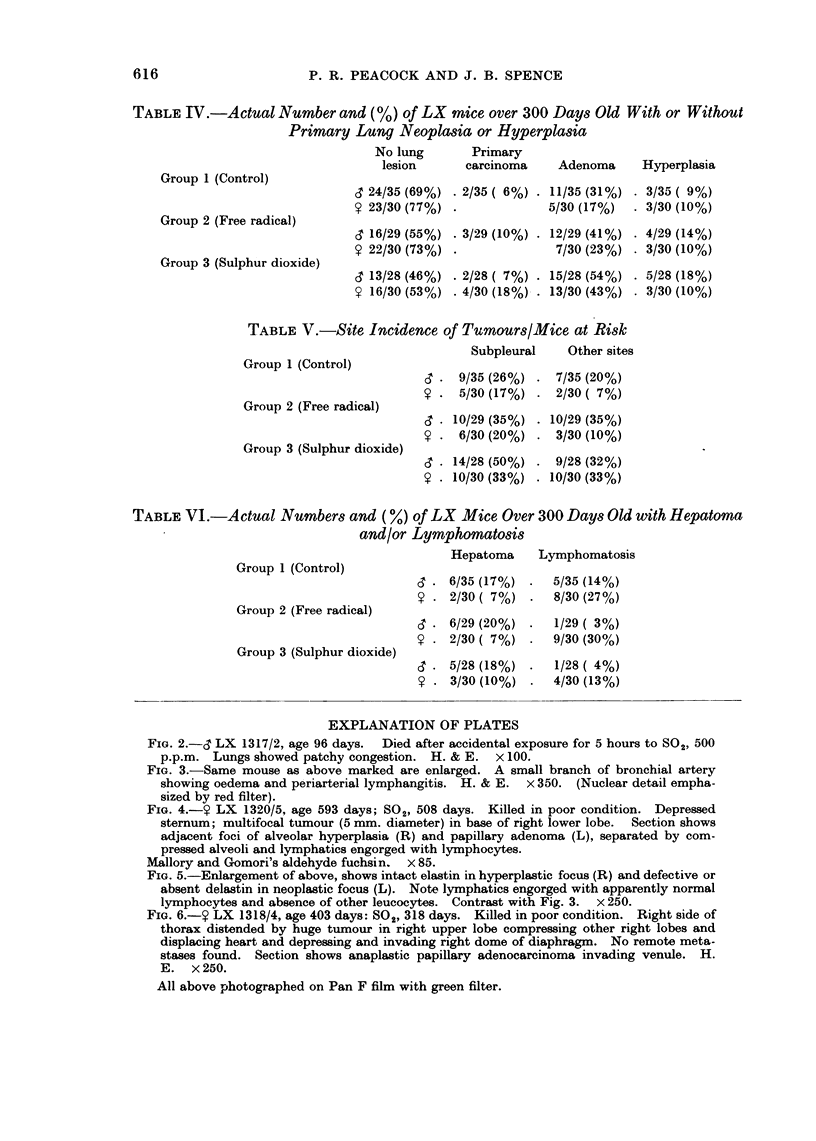

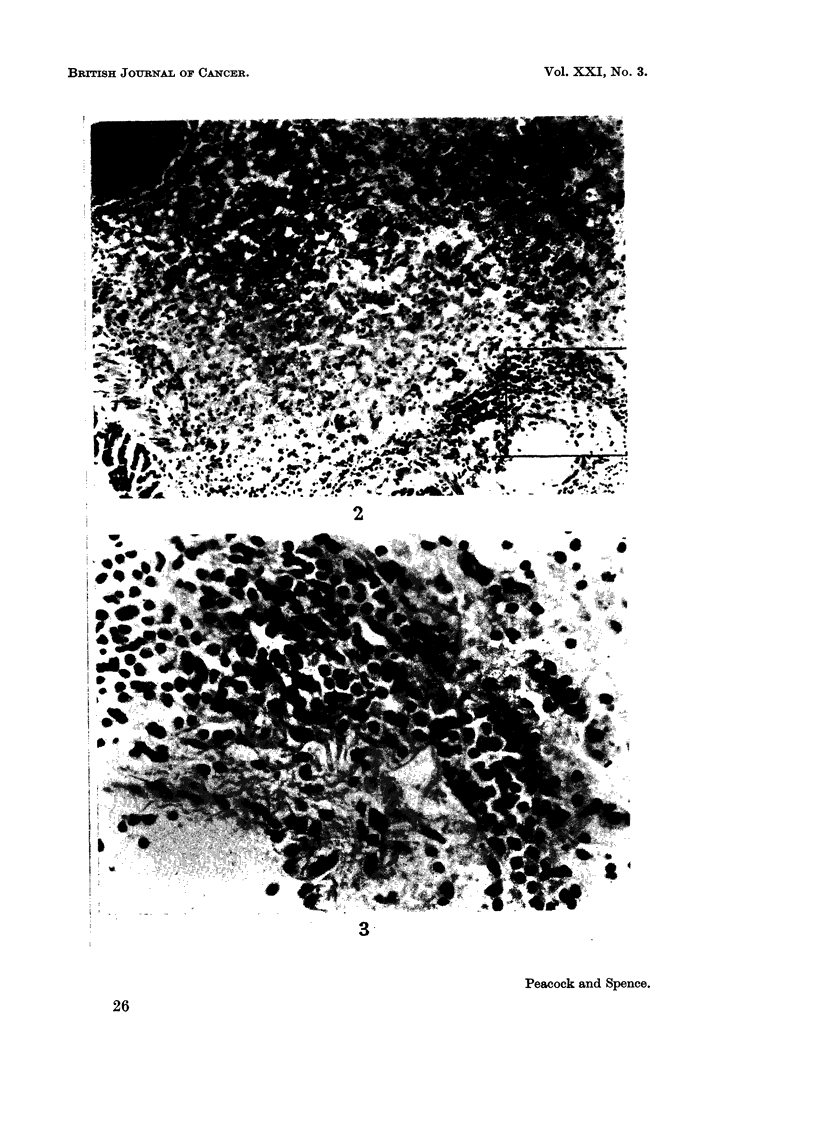

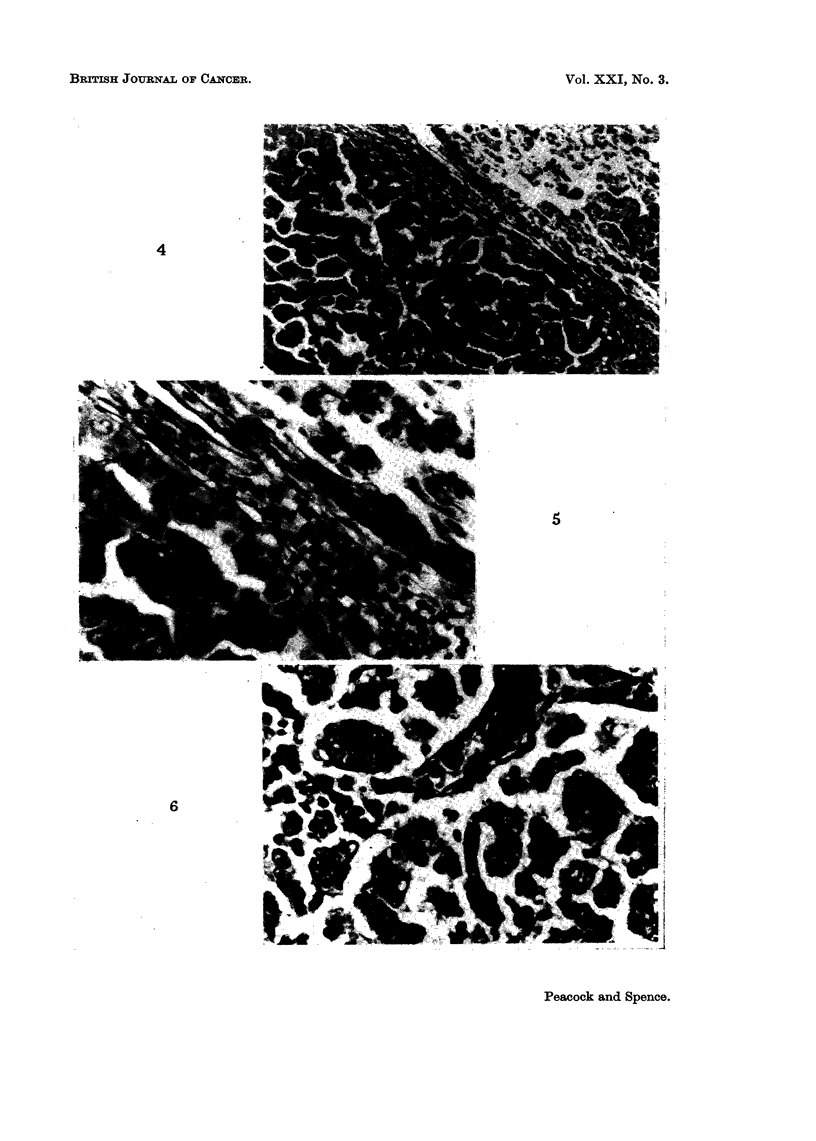

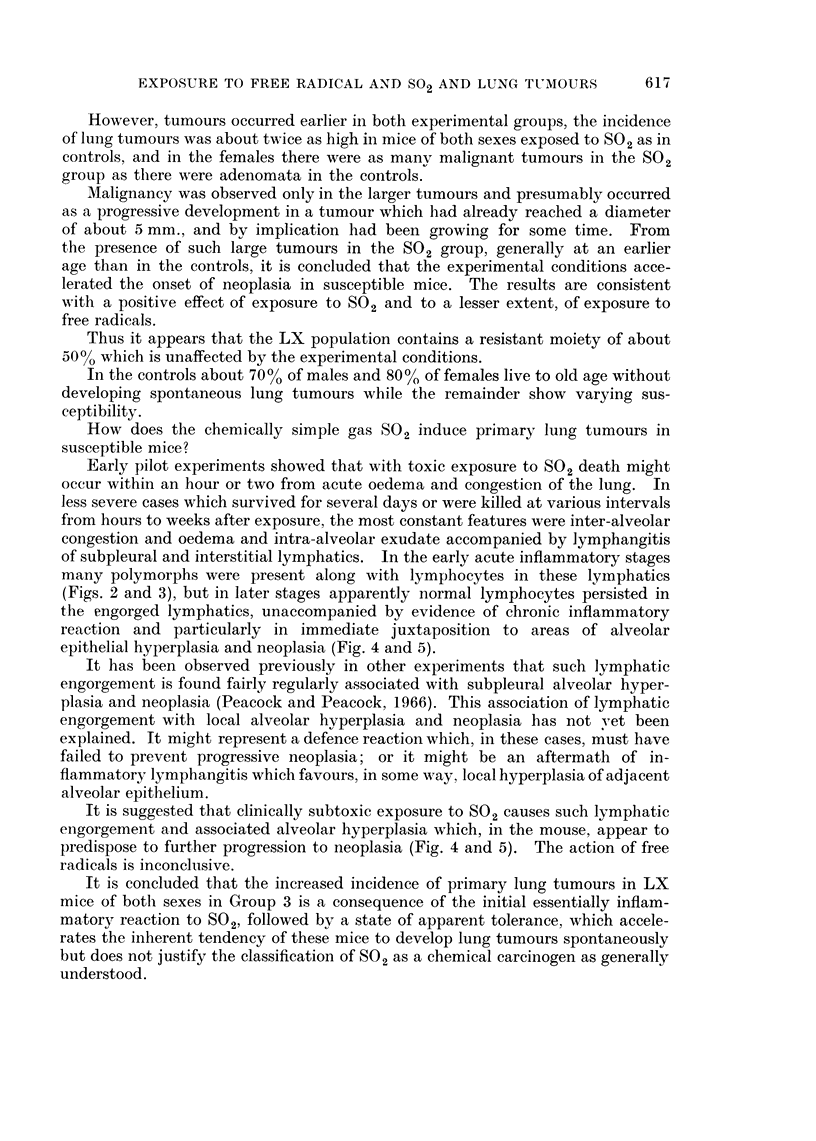

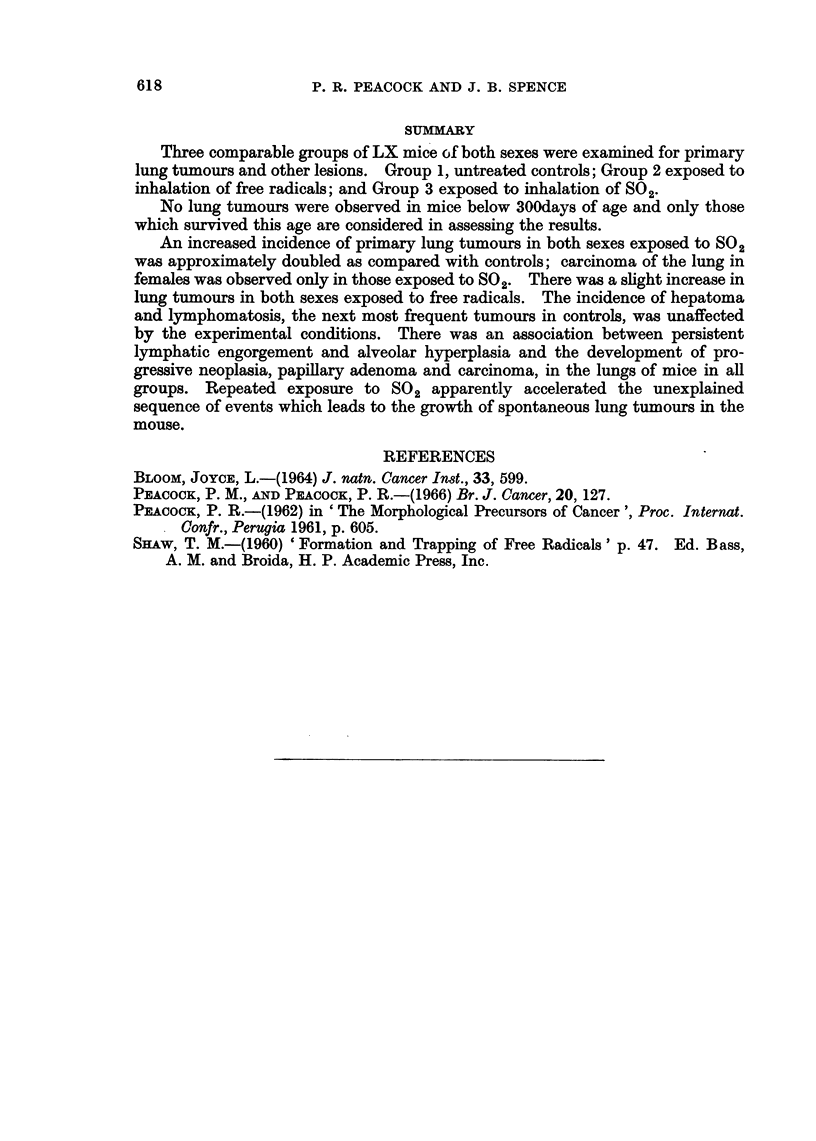

